# Characterising neural plasticity at the single patient level using connectivity fingerprints

**DOI:** 10.1016/j.nicl.2019.101952

**Published:** 2019-07-23

**Authors:** Natalie L. Voets, Oiwi Parker Jones, Rogier B. Mars, Jane E. Adcock, Richard Stacey, Vasileios Apostolopoulos, Puneet Plaha

**Affiliations:** a Wellcome Centre for Integrative Neuroimaging, FMRIB, Nuffield Department of Clinical Neurosciences, University of Oxford, Oxford, UK; b Department of Neurology, Oxford University Hospitals NHS Foundation Trust, John Radcliffe Hospital, Oxford, UK; c Department of Neurosurgery, John Radcliffe Hospital, Oxford University Hospitals NHS Foundation Trust, Oxford OX3 9DU, UK; d Donders Institute for Brain, Cognition and Behaviour, Radboud University Nijmegen, Nijmegen, the Netherlands

**Keywords:** MRI, Tumour, Functional connectivity, Plasticity, Language

## Abstract

The occurrence of wide-scale neuroplasticity in the injured human brain raises hopes for biomarkers to guide personalised treatment. At the individual level, functional reorganisation has proven challenging to quantify using current techniques that are optimised for population-based analyses. In this cross-sectional study, we acquired functional MRI scans in 44 patients (22 men, 22 women, mean age: 39.4 ± 14 years) with a language-dominant hemisphere brain tumour prior to surgery and 23 healthy volunteers (11 men, 12 women, mean age: 36.3 ± 10.9 years) during performance of a verbal fluency task. We applied a recently developed approach to characterise the normal range of functional connectivity patterns during task performance in healthy controls. Next, we statistically quantified differences from the normal in individual patients and evaluated factors driving these differences. We show that the functional connectivity of brain regions involved in language fluency identifies “fingerprints” of brain plasticity in individual patients, not detected using standard task-evoked analyses. In contrast to healthy controls, patients with a tumour in their language dominant hemisphere showed highly variable fingerprints that uniquely distinguished individuals. Atypical fingerprints were influenced by tumour grade and tumour location relative to the typical fluency-activated network. Our findings show how alterations in brain networks can be visualised and statistically quantified from connectivity fingerprints in individual brains. We propose that connectivity fingerprints offer a statistical metric of individually-specific network organisation through which behaviourally-relevant adaptations could be formally quantified and monitored across individuals, treatments and time.

## Highlights

• Personalised treatment awaits individualised measures of brain adaptation.
• Connectivity patterns from FMRI offer unique “fingerprints” of brain networks.
• Individual brain tumours disrupt the language fluency network in unique ways.
• By fingerprint matching, networks can be tested and visualised in single patients.

## 1 Introduction

In the context of disease or injury, brain networks supporting language show an extensive capacity to reorganise (Turkeltaub et al., 2011). However, a wealth of neuroimaging (Balter et al., 2016) and gold-standard clinical (Penfield and Roberts, 1959) investigations indicate that the brain structures involved in language processing vary widely between individuals. Knowledge of individual differences in brain organisation has long been of interest to guide personalised clinical decision-making (Fornito et al., 2015). Locating essential speech and language sites, for example, is critical to minimise risks of language declines following neurosurgery (Bookheimer, 2007). Evidence from longitudinal studies furthermore indicates that brain regions supporting language may adapt following stroke (Saur et al., 2006) and after surgery for epilepsy (Helmstaedter et al., 2006) or brain tumours (Robles et al., 2008). These results have spurred interest in the potential for markers of brain ‘plasticity’ to optimally time surgical interventions (Duffau, 2014) and to select targets for clinical trials of rehabilitative brain stimulation therapies (Grefkes and Fink, 2014).

A major hurdle to developing these clinical applications is the lack of metrics able to quantify whether – and in what way – language networks of an individual patient have ‘re’-organised. The only current non-invasive method to estimate language reorganisation within one patient's brain is the laterality index (LI). This approach compares task-evoked brain activity - usually the number of activated voxels in functional MRI maps - to determine language ‘dominance’ among pre-selected brain regions in the left and the right hemispheres. However, laterality-based measures of language dominance are limited in key respects. In addition to statistical challenges (Ruff et al., 2008), measures of dominance disregard that a small area of brain activity may nonetheless be critical for language function. Furthermore, the laterality approach is, by definition, insensitive to plasticity shown to occur within the injured hemisphere (Duffau, 2014; Southwell et al., 2016; Turkeltaub et al., 2011). Because the continued functioning of an injured brain appears to involve complex changes in the way widespread brain regions interact (Carrera and Tononi, 2014), measures of more distributed network processing are likely needed to inform brain function and recovery (Cramer et al., 2011).

Functional connectivity methods, which measure signal correlations between brain regions, provide a sensitive approach to identify brain organisation at the network level (Cramer et al., 2011). A particularly powerful application of these methods is to identify so-called ‘connectivity fingerprints’, which are patterns of functional coupling that uniquely characterise individual brain regions (Mars et al., 2011; Neubert et al., 2014; Passingham et al., 2002). In recent years the availability of fast, high-resolution fMRI has made it possible to identify variations in connectivity that are specific to individual brains (Finn et al., 2015; Tavor et al., 2016) and inform behaviour (Smith et al., 2015). However, despite these advances, connectivity fingerprinting has not yet been translated into clinical practice due to the need for a practical tool to evaluate if one patient's fingerprint differs from ‘normal’. Analysis methods have recently been developed to formally calculate similarities in connectivity fingerprints across brain regions and between individuals of different species. Such fingerprint comparisons have shed new light onto homologies and unique specializations among human and non-human primate brains (Mars et al., 2016). Consequently, we predicted that this novel approach to quantify connectivity fingerprints would identify the presence and pattern of network alterations in individually injured brains for a key aspect of language: fluency.

Here, we explored the network fingerprint associated with a well-validated word generation language task in a patient model with focal pathology but largely intact performance: newly diagnosed pre-surgical brain tumours. We identified the task-associated connectivity fingerprints in 44 patients with a tumour in their language-dominant hemisphere who were individually compared to healthy controls. Our aims were to evaluate normal variability in network organisation among healthy individuals, identify pathological fingerprints in individual patients, and explore clinical factors driving fingerprint reorganisation. In our data, we found that approximately 50% of individuals with a language-dominant hemisphere tumour have statistically atypical patterns of connectivity with pars opercularis. The manner in which individuals differed was highly variable, and partially explained by the pathological grade and the location of tumours relative to the functionally-defined (fluency task) network. These results illustrate the potential of fingerprinting in the clinical setting as a metric to visualise and quantify functional network modifications at the individual level. The availability of this metric promises a much-needed, practical framework to help guide treatment interventions and progressively measure their effects on targeted brain networks.

## 2 Materials and methods

### 2.1 Experimental design

This cross-sectional study aimed to investigate a newly-developed functional connectivity fingerprinting metric as a marker of neuroplasticity in the language network of patients with a focal brain tumour. Forty-four fluent English-speaking patients (mean age 39.4 ± 14 years (range 19–72, 22 men)) were prospectively recruited through the Oxford neuro-oncology surgery service. We selected all patients harbouring a focal tumour in the language dominant frontal (*n* = 18), temporal lobe (*n* = 14) or insula (*n* = 12) (Table 1, Supplementary Fig. S3a) from our larger cohort of patients being evaluated prior to surgery during the study period (June 2014–2018). Patients were excluded if they had contraindications to MRI, or prior surgery other than biopsy. Forty-two patients had radiological appearances of a tumour in the left hemisphere. Two patients with a right hemisphere tumour presented with seizures causing speech disruption. Atypical (right-hemisphere) language dominance in these patients was established using functional MRI and confirmed in one patient during awake intraoperative brain stimulation; both these patients were left handed. The data for the two right hemisphere patients were swapped across the y-axis so that the ‘affected’ hemisphere was standardised to be the left hemisphere across all patients. The histopathological diagnoses for all but two patients are detailed in Table 1; 1 patient declined surgery and 1 had a non-diagnostic tissue sample.

**Table 1:** Clinical characteristics of tumour patients.

Clinical variable	Number of patients
Sex (M/F)	22/22
Hemisphere harbouring tumour (L/R)*	42/2
Handedness (R/L/ambidextrous)	38/4/2
Confirmed seizure (Y/N)	32/12
Tumour location	
Temporal lobe	14
Frontal lobe	18
Insula	0
Temporo-insular	6
Fronto-insular	6
Pathology	
Low grade	16
(Oligodendroglioma, WHO grade II)	(7)
(Astrocytoma, WHO grade II)	(8)
(Diffuse glioma, NOS)	(1)
High grade	22
(Anaplastic oligodendroglioma, WHO III)	(2)
(Anaplastic astrocytoma, WHO grade III)	(13)
(Glioblastoma, WHO IV)	(7)
Focal cortical dysplasia	1
Dysembryoplastic Neuroepithelial Tumour	2
Meningioma	1
Unknown (suspected glioma)	2

The 2 tumours occurring in the right hemisphere of left-handed patient with speech symptoms (marked with an *) were hemisphere-flipped to the left hemisphere for all analyses. WHO = World Health Organisation. NOS = not otherwise specified.

Twenty-three healthy, right-handed native-English speaking volunteers (mean age 36.3 ± 10.9 years (range 19–68, 12 men)), age-matched to the patient group (t (65) = −0.94, *p* = 0.353), were recruited to quantify normal language network variability. Healthy volunteers were excluded if they had a prior or current neurologic or psychiatric condition. All participants gave informed written consent to take part in this study, in accordance with the principles of the Declaration of Helsinki. The study was approved by the Oxford-B Research Ethics Committee.

### 2.2 Language task

Participants performed a covert word generation task in the scanner, during which they were asked to silently think of words beginning with a visually presented letter (F, A, S, M) (Parker Jones et al., 2017; Voets et al., 2006). The order in which the letter targets were presented was held constant across all participants. Letters were presented in four blocks of 30 s and alternated with a 30 s visual fixation cross, resulting in a total task duration of 4 min and 18 s including dummy scans. The task was practiced out loud with all participants prior to the scan, using a different set of target letters. Phonemic fluency performance was recorded for 12 / 22 healthy controls and 35/44 patients during prior clinical neuropsychological work-up prior or immediately after completing the scan. The total number of words generated in a minute to the letters F, A and S were summed and converted into z-scores. Fluency scores were within the unimpaired range (z-score ≥1.33) for 29/35 patients (82.9%) and all controls.

### 2.3 Magnetic resonance imaging (MRI)

For functional connectivity fingerprints to have potential as a clinical tool, these should be comparable between different MRI scanners and achievable on clinical systems. To determine generalizability, data were acquired on two Siemens 3 T MRI systems at the University of Oxford FMRIB Centre. The healthy volunteers were divided into two subgroups. Ten were scanned using a clinical-grade Verio system; the other 13 using a state-of-the-art research Prisma system, each equipped with a 32-channel head coil. Among the tumour patients, 24 were scanned using the Verio and 20 on the Prisma system.

Echo planar imaging (EPI) blood‑oxygen level dependent (BOLD) signals were acquired for every participant during fluency task performance. The functional MRI sequence parameters are detailed in *Supplementary methods*. A high-resolution (1 mm isotropic) MPRAGE T1-weighted anatomical image was acquired for co-registration of the task FMRI data.

### 2.4 Task analysis

Single subject FMRI data were pre-processed using FMRIB Software Library (FSL, v5.0) tools (www.fmrib.ox.ac.uk/fsl). Standard pre-processing steps included brain extraction (Smith et al., 2002), temporal filtering at 90s (Jenkinson et al., 2002), motion correction, spatial smoothing at 5 mm full width half maximum, and linear registration to the anatomical scan using boundary-based registration (Greve and Fischl, 2009). Individual participant task activation maps were generated using the general linear model incorporated within FSL's FEAT. Head motion parameters and externally recorded physiological parameters (pulse oximetry and respiratory bellows) were modelled when indicated. The resulting z-statistic images were thresholded at a cluster forming z-threshold of 3.1. Clusters were considered significant for *p* < 0.05, GRF-corrected. Second-level analyses were performed using mixed effects analysis to compare average activation maps between patients and controls.

### 2.5 FMRI laterality index (LI) calculations

To evaluate functional connectivity fingerprints against a routinely used clinical measure, we computed a language LI from every participant's fluency task activation map. In each hemisphere, we created a large fronto-temporo-parietal mask combining the MNI template brain parcellations from the Harvard-Oxford atlas. We aligned the resulting masks to each individual's task activation map and quantified the number of active voxels in the left and in the right hemisphere at a range of statistical levels, as previously described (Adcock et al., 2003; Voets et al., 2006). We previously found that calculating each person's LI at a statistical cut-off 2 z-scores below their maximum fMRI activation offered the best agreement with the clinical ‘gold standard’ Wada test in surgical epilepsy patients (Adcock et al., 2003). We therefore determined every participant's LI, calculated using the formula: LI = (L – R) / (L + R), at this proportional threshold. In the resulting values, −1 indicates complete right hemisphere dominance and +1 indicates complete left hemisphere dominance for this task.

### 2.6 Language network regions of interest

Regions of interest for functional connectivity analyses were derived from an extensive review of language networks in neurologically normal populations (Price, 2010). We selected brain regions based on their recognised roles in core stages of speech production (word retrieval, auditory feedback, planning and performing articulation). Left hemisphere pars opercularis (Pop) in particular plays a core role in normal phonemic fluency (Costafreda et al., 2006). Dorsal pars opercularis is implicated in sequence processing that is not specific to language, while the ventral portion is associated with articulatory planning (see Price (2010) for review). Damage to ventral Pop in the dominant hemisphere results in impaired phonological processing after stroke (Lorca-Puls et al., 2017). We therefore selected left ventral Pop (henceforth “Pop”) as the seed from which to calculate the phonemic fluency-related functional connectivity fingerprint. Sixteen target regions (Table 2) were selected from the distributed speech network, including the middle frontal gyrus/inferior frontal sulcus, dorsal anterior cingulate cortex, posterior superior temporal sulcus, caudate, putamen, anteroventral supramarginal gyrus and ventral premotor cortex in both hemispheres, as well as the left anterior supplementary motor area and right pars opercularis. For the Pop ‘seed’ and each of the ‘targets’, a 5 mm spherical mask was created on the MNI template brain, centred on published coordinates (Table 2, Supplementary methods).

**Table 2:** Functional connectivity mask coordinates.

Region	Hemisphere	MNI coordinates (mm)		
		x	y	z
Ventral pars opercularis	L	−52	16	8
	R	54	18	8
Inferior frontal sulcus	L	−51	25	25
	R	52	26	22
Dorsal anterior cingulate gyrus	L	−6	20	34
	R	8	20	36
Posterior superior temporal sulcus	L	−66	−34	−1
	R	66	−34	−2
Caudate nucleus	L	−12	8	10
	R	14	10	10
Putamen	L	−24	−6	6
	R	24	−4	6
Supramarginal gyrus	L	−52	−34	30
	R	54	−24	28
Ventral premotor cortex	L	−52	4	8
	R	54	4	10
Pre-supplementary motor area	L	−4	7	50

Fingerprints were calculated from the left ventral pars opercularis to each of 16 target regions forming part of the wider speech-related language network. For each brain area, a 5 mm sphere was created, centred on x, y, z mm coordinates selected from the literature on the Montreal Neurological Institute (MNI) template brain. For references detailing the selection of brain regions, please see *Supplementary materials & methods*.

### 2.7 Functional connectivity fingerprint analysis

To measure functional connectivity from Pop, we used a seed-based correlation analysis implemented in FSL (O'Reilly et al., 2010; Voets et al., 2014). For this analysis, the atlas-generated Pop mask was nonlinearly aligned to every participant's task functional MRI time-series in order to extract the average fMRI signal time-series during the fluency task. Next, we calculated in each individual participant's fluency task data the correlation between Pop and the fMRI signal time-course of every other voxel in the brain (Supplementary Fig. S1a). The time courses associated with white matter, cerebrospinal fluid and head motion (extracted from tissue segmentations conducted on each individual's T1-weighted anatomical scan) were regressed out from every participant's analysis to minimise non-tissue related confound effects (O'Reilly et al., 2010). The output correlation maps were normalised using Fischer z transforms.

Fingerprints representing the correlation between Pop and each target region were generated by applying the 16 target masks to the whole-brain correlation map (Supplementary Fig. S1b). We then extracted the z-normalised correlation values, averaged across all voxels within each 5 mm sphere mask for every participant. These mean functional correlation values were plotted in spider plots for the groups of patients and healthy controls (Fig. 2) and individual patients (Fig. 3).

To statistically compare the fingerprint of individuals and groups, we used permutation testing to evaluate the Manhattan Distance between individual and group-averaged fingerprints as described by Mars et al., 2013, Mars et al., 2016. The Manhattan Distance represents the summed absolute difference in functional connectivity values between two fingerprints. Concretely, the average Pop fingerprint of healthy controls is formed by the 16 z-normalised correlation values between Pop and each target region. For any given patient, we can calculate if their 16 correlation values are larger or smaller relative to controls, by calculating the Manhattan distance. The smaller the Manhattan Distance, the greater the similarity in fingerprints. All the fingerprint analysis scripts are freely available as part of the MR Comparative Anatomy Toolbox for Matlab (Mr Cat) and can be downloaded from www.neuroecologylab.org.

Three analyses were conducted. First, we assessed the *consistency* of connectivity fingerprints between our two populations of healthy controls. To test if the fingerprints of the control subgroups differ, we used permutation testing as implemented in the MR Comparative Anatomy Toolbox for Matlab (Mr Cat, www.neuroecologylab.org). We performed 5000 permutations across the two groups. This analysis plots the distribution of the Manhattan distance statistic between both groups and then calculates a) a one-tailed significance criterion, representing the value at which the difference between the fingerprints is larger than would be expected by chance, and b) the actual statistic measured from the data. If the test statistic exceeds the significance criterion, the fingerprints of the groups are considered to differ (Mars et al., 2016).

Secondly, we repeated the group permutation analysis to test whether at the *group level*, the network fingerprint of tumour patients differs from the network fingerprint of healthy controls. This analysis would allow us to evaluate whether our multivariate approach (permutations across all connections in the fingerprint) is more sensitive in general than univariate analyses (comparing single group activation maps or LIs) to detect language network reorganisation across patients.

Finally, we performed *single subject analyses*, comparing each patient's individual fingerprint to healthy controls. Our statistical assumption was that language reorganisation constitutes a special scenario, rather than plasticity occurring by default in all patients. We therefore expected the fingerprint of individual patients to match the normal language network, unless (statistically) shown otherwise. If our assumption is correct, the distance between a patient's fingerprint and the average normal fingerprint would - as a rule - be smaller than expected by chance (i.e. match controls). Instead, if a redistribution of network connectivity has occurred, this should result in a patient's fingerprint no longer matching that of controls. To test this assumption, we calculated the Manhattan distance between every patient's fingerprint and the mean fingerprint of controls for 5000 permutations of the 16 target regions in the fingerprint. We determined how many patients remained a statistical match (indicating a normal language network) and how many patients no longer matched the normal network (which we interpret as language network reorganisation).

We performed a post-hoc classification analysis to determine which Pop connections were more likely to differ in brain tumour patients than in healthy controls. The aim of this exploratory analysis was to infer a set of weights on the fingerprint measures that, in general, predict whether a subject is a healthy control or a patient. We could then investigate which part of the fingerprints drove these classifications (detailed in Supplementary *Classification analysis*).

Potential confounding effects of tumour tissue overlapping with pars opercularis or other fluency network regions were investigated by: determining the magnitude of overlap between each patient's tumour and fingerprint regions of interest, repeating group comparisons excluding patients with overlap, and reporting the impact on fingerprint findings (incidence of typical vs atypical).

### 2.8 Multiple linear regression analysis

**Fig. 1:**
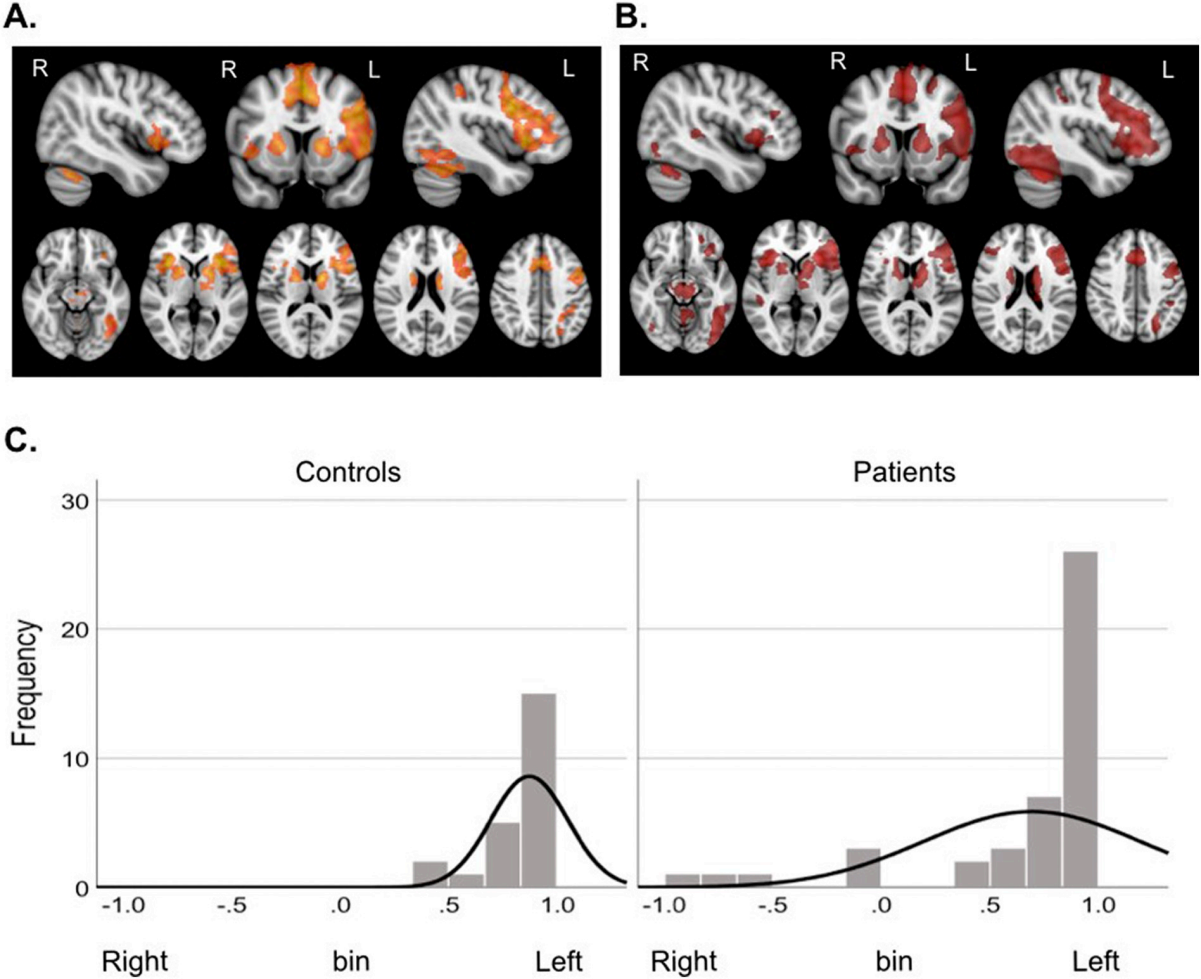
Group fMRI activation maps and LIs during letter fluency. Group mean activation maps in 23 healthy controls (A) and 44 brain tumour patients (B) during a covert fluency functional MRI task. At the group level, both healthy controls and patients activated a typical network of predominantly left-hemisphere brain regions. These included the inferior frontal gyrus (pars opercularis and triangularis), anterior insula, middle frontal gyrus, precentral gyrus, supplementary motor area, anterior cingulate, inferior temporal gyrus and the anterior supramarginal gyrus extending into the superior parietal lobule. Two sample unpaired *t*-tests did not identify any brain regions with greater activation in controls than in patients or vice versa. C. Individual patients had a laterality index (LI) indicative of redistribution of language activation to the contralateral right hemisphere. However, when considered as a group, LIs did not differ between patients and controls (Mann-Whitney test U = 429.5, *p* = 0.303).

Finally, to explore possible causes for a redistributed language networks in patients, we used linear regression analyses to test if clinical variables had a significant relationship with patient fingerprints. Five clinical variables were explored: 1) lesion volume, 2) histopathological grade, 3) history of seizures (in months), 4) volume of overlap (in mm^3^) between the lesion and an anatomically defined mask of ‘Broca's’ area, and 5) volume of overlap (in mm^3^) of the lesion and the language task-activation network measured in controls. Lesion volume was calculated by manually defining the tumour on the T1 anatomical image. Tumour grade was determined from each patient's histopathological report, available in 42 of 44 patients. History of seizures was entered as the number of months since the first (if any) seizure in each patient. Volume of overlap (in mm^3^) between the tumour and Broca's area was calculated by measuring the number of voxels of the manually defined tumour mask that intersected with an anatomically defined mask of Broca's area. The latter was created by summing the pars triangularis and pars opercularis parcellations from the Harvard-Oxford atlas. Volume of overlap (in mm^3^) of the lesion and the healthy controls' language task-activation network was calculated by measuring the number of voxels of the manually defined tumour mask that intersected with the group-average activation map for controls, generated using FSL's FEAT (see *Task Analysis* and Fig. 1a).

### 2.9 Classification analysis

For the classification analysis, we used logistic regression to infer a function that maps from a dataset X (connectivity fingerprints) to a class label y (participant group). The classification analysis is detailed in the *Supplementary methods*.

### 2.10 Statistical analyses

The Shapiro-Wilks test was used to test the normality of selected test variables (age, laterality index and clinical variables) using SPSS (v25). Levene's test was performed to verify homogeneity of variance. When the assumption of normality was violated but Levene's test showed that variances were equal, between-group comparisons were performed using Mann-Whitney tests for non-normally distributed data. For one variable (volume of tumour overlap with the task-evoked language network), Levene's test showed that variances differed between patients with typical and atypical fingerprints. The data were therefore Box-Cox transformed and input to an independent samples *t*-test, reporting the results of the t-test for the case where equal variances are not assumed. Correlation analyses were performed using Spearman's two-tailed correlation coefficient analyses. Multiple linear regression analyses were performed to explore the amount of variance in each connection of patients' Pop fingerprints that was explained by the set of clinical variables. Differences in the incidence of typical vs atypical fingerprints according to tumour location (temporal lobe vs frontal lobe and purely temporal vs purely frontal vs involving the insula) were determined using Chi-square tests.

## 3 Results

To evaluate the use of connectivity fingerprints in a clinical population, we began by identifying limitations of classical measures of neural activation derived from group-level analyses of the same data.

### 3.1 Preserved global network activity

To determine if there is one characteristic, unified neuroplastic response to tumours, we first measured fMRI neural activity from 44 brain tumour patients (Table 1, Supplementary Fig. S3) and from 23 healthy controls, while each performed a silent word generation task. Standard group average maps were contrasted using general linear models to identify differences in brain activity in patients compared to controls. On average, both controls and tumour patients engaged a typical network of left-hemisphere brain regions for the task (Fig. 1a,b). No regions showed statistically greater activation in controls relative to patients or vice versa, at the group level.

This result was confirmed by calculating hemispheric ‘language’ dominance in each individual. The number of statistically activated brain voxels in a large fronto-temporo-parietal region of interest was compared between the left hemisphere and the right in order to generate a laterality index (LI) using our previously-validated approach (Adcock et al., 2003). Healthy controls showed strongly left hemisphere lateralised activation on the covert fluency task, with a mean LI of 0.87 (standard deviation: 0.18, range: 0.41–1). In tumour patients, laterality indices (mean: 0.69) ranged from left (LI = 1) to atypical right hemisphere dominance (LI = −0.86) (Fig. 1c). Six of the 44 tumour patients (14%) had a LI indicative of bilateral or right hemisphere language dominance (namely, between −0.1 and −0.86). At the group level however, there was no difference in LI between patients and controls (Mann-Whitney U = 429.5, *p* = 0.303).

### 3.2 Pathologically altered network connectivity

The wide range of patient LIs in the context of normal group-level results raises two possibilities. The lack of group differences may truly reflect consistent, unaltered fluency-related language networks in the majority of patients. Alternatively, the group findings belie large variability in how networks are affected between patients, obscuring differences in standard analyses that are based only on regional differences in the mean task-evoked neural signal.

**Fig. 2:**
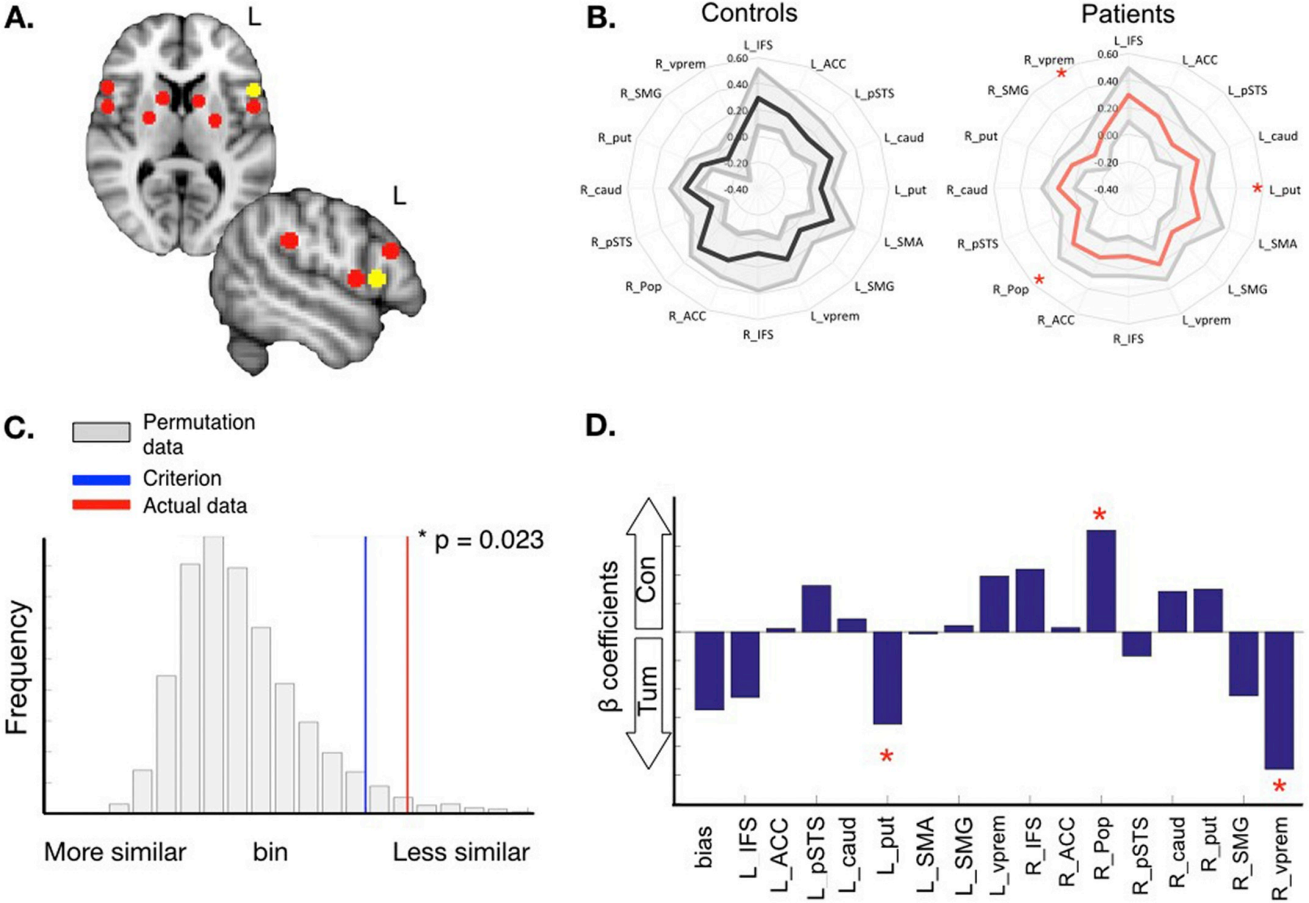
Pars opercularis connectivity fingerprints in controls and tumour patients. A. Signal correlation was measured during silent word generation between left ventral pars opercularis (Pop, yellow mask) and target ‘language network’ regions including contralateral homologues (red spheres, see Table 2) in healthy volunteers (*n* = 23) and brain tumour patients (*n* = 44). Individual subject values were averaged and plotted in spider plots (B) representing Pop connectivity fingerprints for each group. The mean value for each group is represented by the bold line with 1 standard deviation above and below the mean plotted in the grey area. C. The average fingerprints were visually similar, but the distance between patient and control fingerprints (i.e. the difference in fingerprint correlation values) was larger than would be expected by chance (permutation *p*-value = 0.02), indicating a statistical difference between the groups. D. classification analysis identified the left putamen, right pars opercularis and right ventral premotor cortex as significant predictors (*p* < 0.05) of group membership (control vs tumour patient). These are identified by red stars on the bar graph and fingerprint plot. (For interpretation of the references to color in this figure legend, the reader is referred to the web version of this article.)

To test this possibility, we examined connectivity fingerprints measured from left hemisphere ventral pars opercularis (Pop) - a key structure involved in speech production (Price, 2012) - during the fMRI word generation task. For each person, we plotted the strength of signal correlation between Pop and a distributed set of 16 fluency-related language network structures. These regions were objectively selected from the literature and included ipsilateral left hemisphere structures and contralateral homologues (Fig. 2a, Table 2). Before testing patient fingerprints, we established the robustness of normal Pop connectivity fingerprints across two groups of healthy volunteers scanned using different MRI systems and sequences (see *Methods*). Using permutation testing, the distance between fingerprints of the two control groups was not larger than would be expected by chance (*p* = 0.114, Supplementary Fig. S2). In other words, fingerprints of healthy controls did not differ, and were pooled for subsequent patient comparisons. In contrast, the difference between the fingerprints of tumour patients and controls was significantly larger than expected by chance (*p* = 0.023, Fig. 2b,c), indicating a group-wise difference in network organisation that was not apparent from the previous univariate and LI task activation results. A classification analysis, using all 16 branches of the fingerprint as input, identified the ipsilateral putamen, right pars opercularis and right ventral premotor cortex as significant predictors (*p* < 0.05) of group status as a tumour patient or a control (Fig. 2d).

**Fig. 3:**
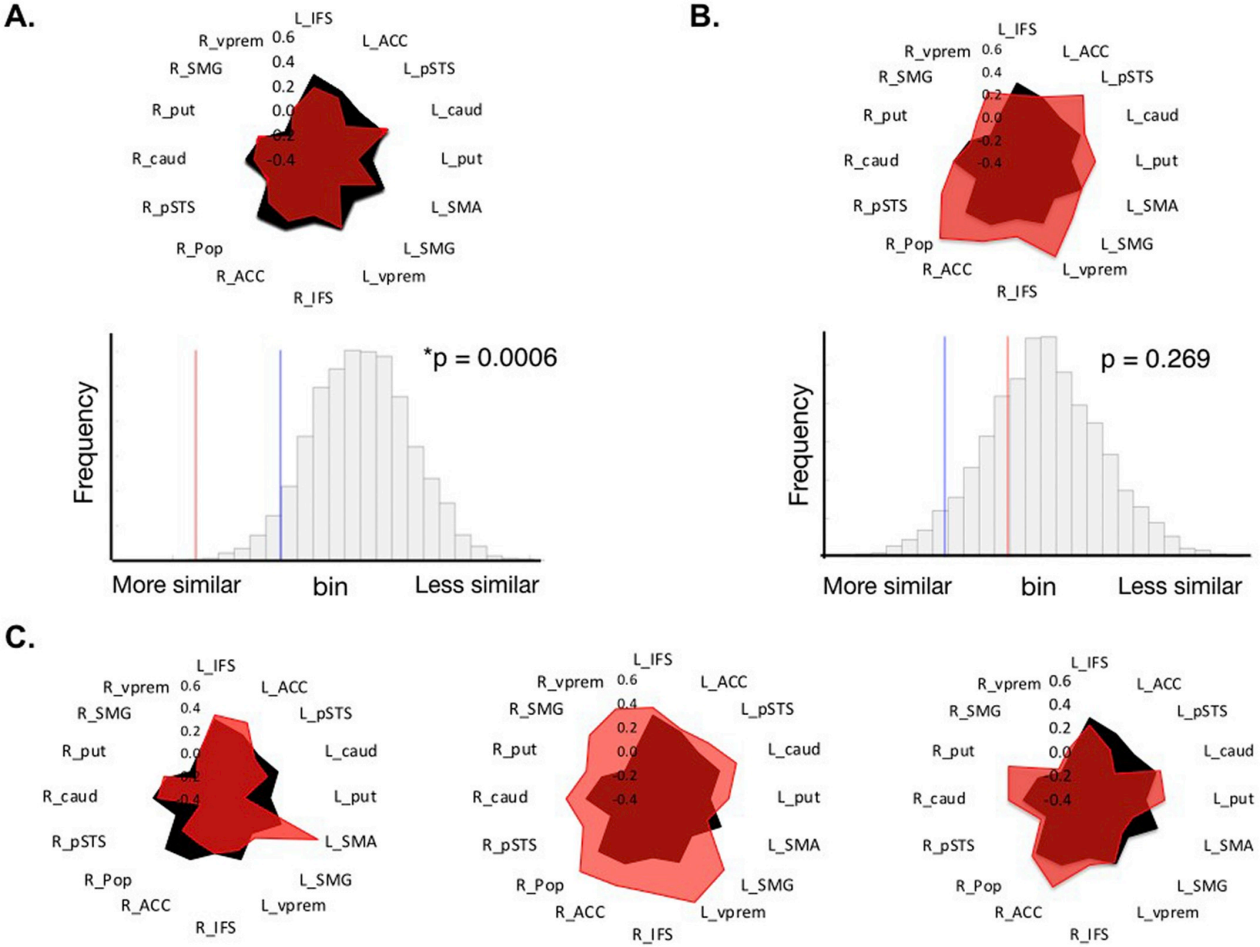
Individual variation in patient connectivity fingerprints. Comparing the functional connectivity fingerprint of individual patients to the template fingerprint of controls identified a statistical match in some patients (A), but not in others (B). In both cases, the fingerprint of the individual patient (red) is overlaid onto the average fingerprint of all controls (black) and shown alongside the result of statistical permutation tests. In patient A, the difference between the patient's fingerprint and that of the healthy controls (observed test statistic, red line) is smaller than expected by chance (criterion value, blue line), indicating a statistical match between the two fingerprints (permutation p-value = 0.0006). In patient B, the difference did not pass the criterion value, i.e. this patient's fingerprint was not a match to controls (*p* = 0.27). C. Three individual patients whose fingerprints statistically deviated from ‘normal’ in unique ways, including globally increased functional connectivity or heightened connectivity to specific brain structures. (For interpretation of the references to color in this figure legend, the reader is referred to the web version of this article.)

Next, to determine if fingerprints detect atypical fluency networks in *individual patients*, we evaluated each patient's fingerprint independently against healthy controls. In 22 / 44 (50%) patients, the distance between their Pop fingerprints and that of controls was smaller than expected by chance (i.e. matched the normal network, p < 0.05). The remaining 22 patients had a connectivity fingerprint that did not match controls. In comparison, none of the individual control fingerprints were significantly different from the rest of the control group (using a leave one out analysis). Representative results are shown in Fig. 3. Individual patients deviated from the normal network in a number of distinct ways. Some patients showed localised changes in Pop functional connectivity involving ipsilateral and/or contralateral regions of the network relative to controls. Other patients showed global deviations along most branches of the fingerprint. Importantly, by formally testing each patient's fingerprint independently, these unique variations could be quantified statistically. In this way, fingerprints indicated explicitly *how* a single patient's network deviated from normal, and uncovered unique variance among the patients that was hidden in group-level analyses.

These variations in patient fingerprints were not explained by the presence of tumour tissue within the Pop or network target areas. If a patient's tumour overlapped directly with Pop, a possible confound might arise whereby the whole ‘language’ network would look abnormal because pathological (non-neuronal) Pop signal was measured. To test this possibility, we calculated how many patients' tumour overlapped with the Pop mask. In 8/44 patients (18.2%), the tumour overlapped at least partially with the Pop connectivity mask. The overlap was minimal (<3%) in 2 patients, but substantial (ranging from 57 to 100% overlap) in the other 6. Nevertheless, the results of the group comparison between healthy controls and patients were unchanged after excluding these 8 patients: the difference between the fingerprint of tumour patients and controls remained significantly different (*p* = 0.034). Importantly, at the individual patient level, atypical fingerprints were not driven by tumour tissue within Pop. Only 5/22 (22.7%) patients with an atypical fingerprint had tumour overlapping with Pop, while 3 patients' fingerprints (13.6%) were statistically normal despite comparable levels of overlap.

A second possibility is that patient fingerprints might deviate only in specific regions of the network selectively affected by tumour. This result would not constitute a confound per se, but implies that an abnormal fingerprint could be anticipated based on the anatomical location of a tumour, without needing to measure the connectivity fingerprint. This possibility was not suggested by our individual patients results (Fig. 3), which instead indicated frequently widespread network alterations involving contralateral structures that were never affected by tumour. Nevertheless, in the 22 patients who had an atypical Pop fingerprint, we verified if their tumour overlapped with any of the 8 network target masks that were also in the left hemisphere. Seven patients (31.8%) showed no overlap between their tumour and any network target region (nor with Pop). An additional 2 patients had <1% overlap. In the remaining 13 patients (59%), tumour overlapped with at least one of the network targets. In these 13, we tested if the remaining Pop fingerprint remained atypical (i.e. ‘non-match’ to controls) after removing those connections affected by tumour tissue. In 2 patients, the Pop fingerprint changed from atypical to normal after excluding the tumour-affected network connections. Fingerprints of the remaining 11 patients (90.9%) remained atypical. Therefore, tumour tissue alone explained an atypical fingerprint in <10% of this group and factors other than tumours tissue alone drive fingerprint variance among patients.

### 3.3 Predicting atypical fingerprints from clinical characteristics

We next examined if variability in patient fingerprints was predicted by specific clinical characteristics, using linear regression analyses. There was no difference in the incidence of typical vs atypical fingerprints between patients with a temporal lobe or frontal lobe tumour (*χ* (1) = 1.47, *p* = 0.23, Supplementary Fig. S3b), or between patients with a temporal, frontal or insula-involving tumour (*χ* (2) = 4.13, *p* = 0.13). Age, tumour volume and history of seizures were not correlated with Pop functional connectivity (all *p* > 0.05), and therefore not considered further in the regression model. Instead, histopathological grade and tumour location (both in terms of overlap with anatomically-defined ‘Broca's’ area, and of overlap with the functionally-defined task-activated language network) were significant predictors of variability in patient fingerprints. Together, these variables accounted for up to 25% of Pop functional connectivity (Table 3). Lesion overlap with the normal task-activated language network was the most consistent contributor to fingerprint variability. Greater overlap was associated with reduced Pop functional connectivity with the ipsilateral ventral premotor cortex (*β* = −0.77, *p* = 0.003). Greater overlap was also associated with heightened connectivity with the supramarginal gyrus bilaterally (ipsilateral: *β* = 0.804, *p* = 0.004, contralateral: *β* = 0.865, p = 0.003) as well as with contralateral pars opercularis (*β* = 0.619, *p* = 0.037) and ventral premotor cortex (*β* = 0.632, *p* = 0.032). Finally, higher tumour grade contributed to reduced Pop – left caudate connectivity (*β* = −0.404, *p* = 0.011). The distribution of atypical and typical fingerprints according to tumour histological grade is illustrated in Supplementary Fig. S3c. Interestingly, of these three variables, only the amount of tumour overlap with the normal task-activated network significantly differed when directly contrasting patients with typical (*n* = 22) versus atypical (n = 22) fingerprints. A higher amount of lesion overlap occurred in patients whose fingerprints did not match the normal network (independent samples *t*-test, *t* (34.5) = −2.1, *p* = 0.043).

**Table 3:** Variance in patient fingerprints explained by clinical variables.

Fingerprint target region	Variance explained (R^2^)	Model	
		F (regression df, residual df)	p
L vprem	0.238	3.95 (3,38)	0.015
R SMG	0.230	3.79 (3,38)	0.018
L caud	0.245	4.12 (3,38)	0.013
L SMG	0.242	4.04 (3,38)	0.014

Multiple linear regression analysis results indicating the amount of variance in pars opercularis (Pop) functional connectivity explained by a combination of 3 clinical variables in brain tumour patients: histological grade, volume of lesion overlap with anatomically-defined “Broca's area” and volume of lesion overlap with the healthy control task-activated fluency network. Functional connectivity between Pop and four network regions was significantly predicted by the clinical characteristics of individual patients (all p < 0.05). The coefficients for individual clinical predictors are detailed in the Results. L/R = left/right hemisphere. Vprem = ventral premotor cortex. SMG = supramarginal gyrus. Caud = caudate nucleus.

## 4 Discussion

The potential to modulate plasticity for therapeutic gain has received increasing attention to maximise recovery (Otal et al., 2015) (although see Elsner et al. (2015)), and to stage (Duffau and Taillandier, 2015) and monitor the effects of treatment interventions (Kent et al., 2009; Woodhead et al., 2013). Understanding the impact of pathology on network organisation therefore holds promise to identify avenues to promote functionally-relevant plasticity. Yet to date, knowledge that brain plasticity occurs has had relatively little impact on tailoring treatment, because evidence from large patient cohorts have limited ability to inform responses in the uniquely individual brain. Here, we explored the use of connectivity fingerprints as a way to visualise and compare functional connectivity profiles at the individual level in patients with a brain tumour. We show that individually-unique patterns of network organisation can be quantified from connectivity fingerprints in single patients and offer greater sensitivity to fluency-related network alterations than the laterality index. These results illustrate the advantages of connectivity fingerprints in the clinical setting. These advantages are (1) the ability to visualise complex data in a readily interpretable way and (2) a method for measuring whether individual connectivity profiles (fingerprints) are statistically altered from the norm. We further demonstrate the potential of this tool to uncover the influence of clinical factors, such as tumour grade, on functional network re-organisation. We propose that connectivity fingerprint matching has potential direct future applicability to test and monitor clinically relevant brain network adaptations during treatment interventions.

Dynamic changes in brain network organisation are characteristic of the adaptive brain and accentuated under pathological circumstances (Holtmaat and Svoboda, 2009). Electrical stimulation studies in patients undergoing awake surgery first highlighted extensive individual variability in the location of cortical language sites (Ojemann et al., 1989; Penfield and Roberts, 1959). The few existing group activation (positron emission tomography and functional MRI) studies, however, report mixed findings in relation to systematic language plasticity among tumour patients. Some studies found evidence for reorganisation (Partovi et al., 2012; Thiel et al., 2001; Wang et al., 2013), while others did not (Schlosser et al., 2002). Inconsistent findings between studies likely reflect, among other factors, wide variability between patients. Previous studies suggest approximately 30% of right-handed patients with epilepsy and brain tumours have atypical right-hemisphere language dominance (Bauer et al., 2014). An additional 12–35% of patients may have redistributed activity within the affected hemisphere (Mbwana et al., 2009; You et al., 2011). Such intra-hemispheric reorganisation has to date been challenging to quantify (Rosenberger et al., 2009). This is in part because established fMRI analyses are optimised to compare task-evoked signal between groups or experimental conditions, rather than to determine what is normal versus abnormal for one individual.

By formally evaluating connectivity fingerprints of a well characterised speech network, we identified differences in network organisation that uniquely characterised individuals. Numerous patients demonstrated increased or decreased coupling with specific brain regions, whereas others showed up-regulated network connectivity as a whole. This high heterogeneity mirrors and extends upon previous studies that identified at least 9 different variants of language laterality in patients with chronic epilepsy (Berl et al., 2014; Mbwana et al., 2009). However, previous attempts to characterise patterns of language plasticity have typically relied on ‘clustering’ approaches to identify subgroups of patients within a larger population. The results of clustering approaches depend upon who else is included in the study. Connectivity fingerprints, instead, offer an alternative unbiased and practical way to assess individual patients, irrespective of the wider clinical population. Using this approach, we could distinguish not only whether fingerprint changes had occurred, but also in what way a given patient differed from the archetypal network, at least in terms of the strength of connectivity among typical network regions. Plasticity could, of course, also be reflected in a spatial re-distribution of network connectivity. While we would expect that spatial reorganisation might alter the strength of connectivity among typical network regions, further investigation is needed to assess network adaptations outside of the established fluency network. In this respect, it is important that we found that neurologically normal fingerprints were consistent across very different MRI datasets (acquired on two MRI scanners). If this result generalises to other populations and cognitive networks, the normal distribution for fingerprint comparisons could in principle be generated from any control dataset, including for example a publicly available database such as the Human Connectome Project and evaluated comprehensively with much larger datasets.

The single most consistent predictor of altered Pop network connectivity was the location of a tumour relative to the functional, task-evoked speech production network in healthy controls. This finding is largely consistent with prior observations indicating the influence of lesion location on language lateralisation (Partovi et al., 2012; Wellmer et al., 2009). Conversely, tumour volume was not correlated with language network (re)organisation, in keeping with previous studies of patients with various surgical (predominantly tumour) pathologies (Benson et al., 1999; Thiel et al., 2001). While we anticipated that a history of seizures might impact on network organisation, the link between epileptic activity and language plasticity, at least in the adult brain, remains unclear. Seizure history was not indicative of network reorganisation in our study, and seizure frequency was not associated with language reorganisation in a previous study of chronic epilepsy patients (Janszky et al., 2006). However, our tumour patients often presented with a single seizure, limiting any simple parallels that can be drawn with chronic epilepsy. Instead, histopathological grade was a predictor of connectivity between left hemisphere Pop and the ipsilateral caudate, but did not account for increased contralateral coupling. Some groups have proposed that slow-growing tumours have a greater capacity to induce network reorganisation than fast growing tumours (Duffau and Taillandier, 2015; Thiel et al., 2006). The distinction between slow- and fast-growing tumours typically follows histopathological grading, dividing into tumours of World Health Organisation (WHO) grades I-II versus III-IV. However, the stratification of brain tumours has recently been refined, recognising genetic variants that are associated with a more favourable (i.e. prolonged) disease course independent of grade (Louis et al., 2016). The distinction between low- and high-grade tumours may, therefore, be misleading in the context of their potential to induce brain reorganisation. Instead, molecular and biochemical subtyping offer an avenue for further research, since the amount of fingerprint variance explained by typical clinical factors was limited to 25%. An additional possible contributor to neuroplasticity that merits further investigation is the impact of tumour growth on long-range white matter pathways. Given that the functions of a brain region are thought to be informed by its inputs (Honey et al., 2010), damage to specific anatomical white matter tracts underlying language processing (Tzourio-Mazoyer et al., 2017) may plausibly modulate language network adaptation and predict its behavioural impact (Herbet et al., 2016). Newly developed metrics of inter-regional structural connectivity (Mars et al., 2018) may offer additional insight into deviations in functional connectivity fingerprints.

Discovering the numerous ways in which a functioning network may be organised is a key step to understanding brain recovery (Price and Friston, 2002). Following stroke, progressive changes in local brain activity track improvements in language performance at difference stages of recovery (Saur et al., 2006). Nonetheless, while we refer to altered fingerprints as a marker of ‘language’ plasticity, several regions within the extended speech network *support* but are *not specific* for language (Geranmayeh et al., 2014). Additionally, widespread cellular and molecular changes in the injured brain (Zatorre et al., 2012) suggests that plasticity is not automatically relevant to function. Furthermore, the contribution of individual network regions to language in the pathological brain may depend on the stage of impairment (Hartwigsen and Saur, 2017). Our patients were diagnosed following a seizure or transient episode of speech disturbance, but were largely language intact. Consequently, precise behavioural interpretation of changes in network topology will require further validation work, including in patients with a range of performance abilities and using additional tasks probing different aspects of language function.

Further validation will be needed to enable connectivity fingerprinting to realise full potential in guiding treatment planning and monitoring. Our focus was on demonstrating the feasibility to detect network variations at the individual level among a reasonably-sized group of tumour patients, as the lack of robust individual-specific markers is a primary hurdle in clinical translation. Because brain tumours are rare, additional characterisation of fingerprints through multi-site studies across large cohorts of patients will clearly be important to fully elucidate the impact of tumour subtypes and histopathological features on network organisation. Additionally, we focussed on a task of speech production that has been extensively validated and offers high ecological validity, since impairments in generating speech have a dramatic impact on quality of life. Nonetheless, it will be important to evaluate the sensitivity of network metrics to other aspects of language among a wider range of individuals to assess the impact bilingualism, handedness, and other prevalent, clinically relevant factors on ‘typical’ fingerprints. For example, it has been shown that some language functions, such as reading, may be performed using different strategies involving separable neural routes (Seghier et al., 2012). It is currently challenging to distinguish whether variations in network connectivity reflect a re-balancing of functional roles between brain structures or the use of alternate strategies. Resolving this distinction will require very large samples from collaborative initiatives to determine the range of alternative information processing routes for any one brain network in the normal population. Finally, longitudinal studies investigating both the time-frame and the magnitude of fingerprint changes across interventions will be important to inform clinical trial designs incorporating fingerprints as outcome measures of plasticity.

Despite these future challenges, we have shown for the first time that connectivity fingerprints offer a sensitive, statistically objective and clinically-informative method to quantify alterations in brain networks in individual brains. The formal framework within which individual fingerprints can be evaluated offers a practical, key step towards personalised decision-making. This approach, if it proves generalizable across brain networks and disease states, could have wide-ranging use to guide and monitor therapeutic interventions spanning across neurosurgical, pharmacological, behavioural and neuromodulation-based treatments.

## Declarations of Competing Interests

None.

## Appendix A Supplementary data

Supplementary material Image 1
