# Supplementary material for: Characterising neural plasticity at the single patient level using connectivity fingerprints
Source: Neuroimage Clin. 2019 Jul 23;24:101952. doi: 10.1016/j.nicl.2019.101952 (PMC6664196; doi:10.1016/j.nicl.2019.101952)
Supplement: Supplementary file 1 — Supplementary material [file mmc1.docx]

**Supplementary Materials and methods**

**Language network regions of interest**

Regions of interest for functional connectivity analyses were derived from an extensive review of language networks in neurologically normal populations (Price, 2010), references cited therein and further literature searches.

The ‘seed’ region for functional connected analyses was the left hemisphere ventral pars opercularis (Pop), which has a critical role in word generation and phonological processing (reviewed in (Price, 2010)). The coordinates for left Pop were selected on the MNI template brain at location [-52 16 8]. This site was selected from an analysis of brain regions associated with phonological errors elicited by transcranial magnetic stimulation in healthy volunteers, and after stroke in patients (Lorca-Puls *et al.*, 2017), cross-referenced against sites associated with articulatory errors during intraoperative awake direct brain stimulation (Tate *et al.*, 2014).

Sixteen ‘target’ brain regions (Table 2, main manuscript) were selected for Pop functional connectivity computations based on their recognised roles in core stages of speech production (word retrieval, auditory feedback, planning and performing articulation).

Target area 1. The left hemisphere middle frontal gyrus (MFG) / inferior frontal sulcus (IFS) junction is systematically activated in brain imaging studies of speech production and thought to play a role in word retrieval (Price, 2010). The coordinates [-51 25 25] for this region were selected from functional MRI (fMRI) studies that attempted to isolate word generation from reading and show consistent activation for word retrieval (Jeon *et al.*, 2009).

Target 2: The left hemisphere dorsal anterior cingulate cortex (ACC) is thought to play a role in response selection among competing options, i.e. suppressing unwanted words (Price, 2012). MNI coordinates for this region [-6 20 34] were chosen from an fMRI study of error detection during internal speech (Gauvin *et al.*, 2016).

Target 3: The left hemisphere posterior superior temporal region (classically defined as ‘Wernicke’s area’) is a functionally heterogeneous region (Wise *et al.*, 2001) ascribed a role in speech processing and repetition. Coordinates were defined at [-66 -34 -1], approximating a region showing joint activation to word perception and retrieval in a PET study (Wise *et al.*, 2001).

Target 4: The left hemisphere caudate nucleus was selected based on evidence of perseverative errors following stroke and direct brain stimulation (Gil Robles *et al.*, 2005). The centre of the region of interest was defined at coordinates [-12 8 10], chosen to visually approximate the location of intraoperative speech errors identified in a case report (Gil Robles *et al.*, 2005).

Target 5: The left hemisphere putamen was selected based on its purported involvement in articulation (Price, 2010). Selected coordinates [-24 -6 6] were based on those from a representative study contrasting speech production with perception (Fridriksson *et al.*, 2009).

Target 6: The left hemisphere pre-supplementary motor area (pre-SMA), is involved in the initiation of speech. Surgical removal of the pre-SMA induces varying degrees of speech disruption (Laplane *et al.*, 1977; Krainik *et al.*, 2003). Coordinates for the pre-SMA region of interest were centred on [-4 7 50].

Target 7: The left hemisphere supramarginal gyrus (SMG) has been repeatedly implicated in feedback monitoring during speech. The SMG, however, is a functionally heterogeneous region (Oberhuber *et al.*, 2016). We, therefore, selected the SMG target area [-52 -34 30] based on coordinates associated with phonological impairments during transcranial magnetic stimulation or after stroke (Lorca-Puls *et al.*, 2017).

Target 8: The left hemisphere ventral premotor cortex was selected based on the frequent occurrence of speech arrest during intra-operative direct brain stimulation in brain tumour patients (Tate *et al.*, 2014). Coordinates were centred on [-52 4 8].

Target 9: The homologue right hemisphere MFG/IFS, defined at [52 26 22].

Target 10: The homologue right hemisphere dorsal ACC, defined at [8 20 36].

Target 11: The homologue right hemisphere Pop, defined at [54 18 8].

Target 12: The homologue right hemisphere pSTS, defined at [66 -34 -2].

Target 13: The homologue right hemisphere caudate nucleus, defined at [14 10 10].

Target 14: The homologue right hemisphere putamen, defined at [24 -4 6].

Target 15: The homologue right hemisphere SMG, defined at [54 -24 28].

Target 16: The homologue right hemisphere ventral premotor cortex, defined at [54 4 10].

**Functional MRI sequence parameters**

Echo planar imaging (EPI) blood-oxygen level dependent (BOLD) signals were acquired for every participant during fluency task performance. The functional MRI (fMRI) sequence parameters on the Verio scanner were: TR = 3000ms, TE = 28ms, voxel size = 3 × 3 × 3mm, GRAPPA acceleration factor 2, FOV = 192mm^2^, matrix size = 64, 81 volumes, for a duration of 4 minutes and 12 seconds. On the Prisma system, fMRI data were acquired using a simultaneous multislice acquisition sequence, with parameters: TR = 933ms, TE = 33.4ms, voxel size = 2 × 2 × 2mm, multiband acceleration factor = 6, FOV = 192mm^2^, matrix size = 96, 267 volumes, for a total duration of 4 minutes and 18 seconds. A high-resolution (1mm isotropic) MPRAGE T1-weighted anatomical image was acquired for co-registration of the task FMRI data.

**Classification analysis**

For the classification analysis, we used logistic regression. With logistic regression, we infer a function that maps from a dataset **X** to a class label **y**. In our case **X** initially corresponded to a 66 × 16 (subject-by-fingerprint) matrix and **y** to a 66-dimensional (i.e. subject-length) vector of one-bit integers with values corresponding to ‘healthy control’ or ‘patient’ groups. We pre-processed the data in **X** by subtracting the mean and then dividing standard deviations for each element column-wise, in order to normalise the distribution of fingerprint values to have a mean of 0 and unit variance. We then added a column of 1s to **X** to model group bias. These are standard pre-processing steps not only for logistic regression but also for many other varieties of supervised learning. Because the final shape of **X** was 66 × 17 (i.e. 66 subject rows × 16 fingerprint measures + 1 intercept column), we randomly initialised a 17 × 1 weight matrix, **W**, drawn from a Gaussian distribution with mean 0 and unit variance. For each subject, we can define a forward pass through our model that will map from 17 input features to 1 output label. We used batch gradient descent with a cross-entropy loss to infer an optimal set of model weights, **W**. A learning rate of 0.01 was selected from prior experience and the model was trained until the loss converged. We then inspected the optimised weights (or beta coefficients) in **W**. We did not split the data before training as is commonly done, because we were not interested in the generalisability of the model but rather in exploring an optimised set of weights, which best explain group membership over all subjects. Two facts about the data and coding when interpreting the optimised weights. First, the fingerprint scores were positive or negative correlations between ROI time-series, and second, we coded patients as 0 and controls as 1 in the target vector, **y**. This means that positive weights on positive input correlations predict controls and negative weights on positive input correlations predict patients. If the input correlations are negative, then the reverse is true: positive weights will predict patients and negative weights will predict controls. Teasing apart the specific contribution of a weight can therefore be knotty, but resolvable. Because the weights correspond to known connections between left Pop and other regions of interest, we can use the model weights to gain insight into which connections, in general, drive the classification of subjects into their respective groups. We note that since there were more patients than controls in our sample, we expected the bias weight to be negative, that is, in the direction of the 0 (as opposed to the 1) encoding of **y**.

**Supplementary results**

**Supplementary Fig. S1.**

**
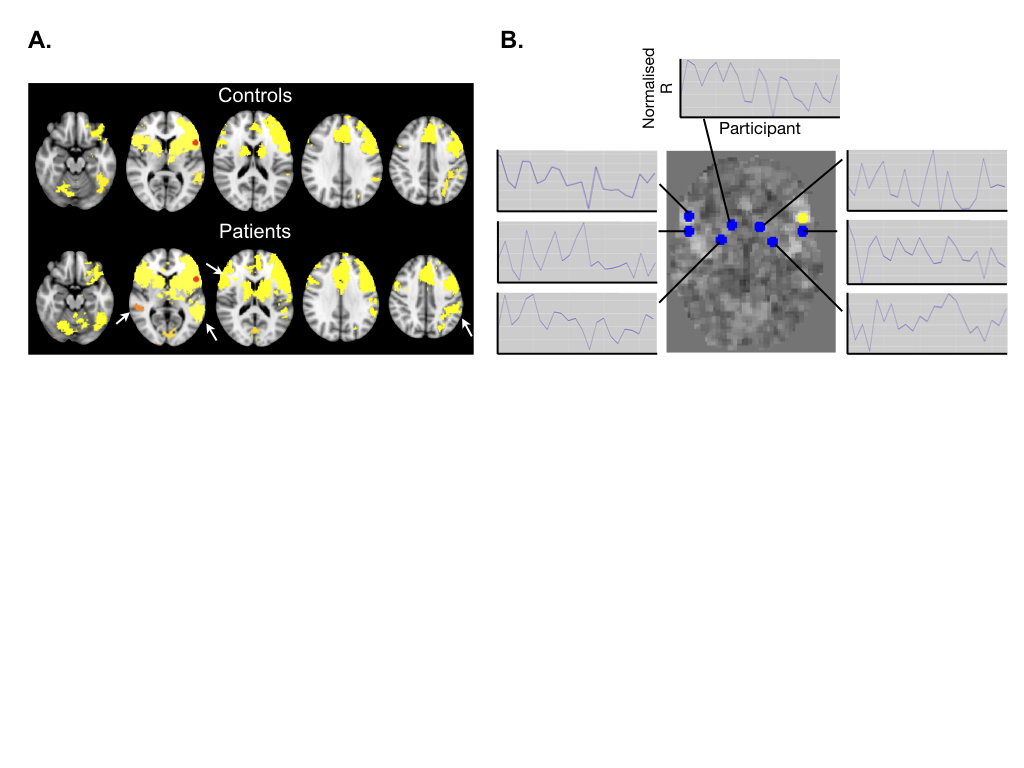
**

**Connectivity fingerprint analysis process. A**. Functional connectivity was measured from left pars opercularis (Pop, red sphere) to the rest of the brain in healthy controls (n=23) and brain tumours patients (n=44) during performance of a covert fluency task. Both groups showed functional coupling between Pop and a widespread network of brain regions, which qualitatively appeared enhanced in some regions in patients (white arrows). **B**. For quantitative connectivity fingerprint analyses, the average signal correlation with Pop was extracted from 16 published language network regions (representative blue spheres, see also Table 2 in the main manuscript) and their contralateral homologues in all individuals.

**Supplementary Fig S2.**

**
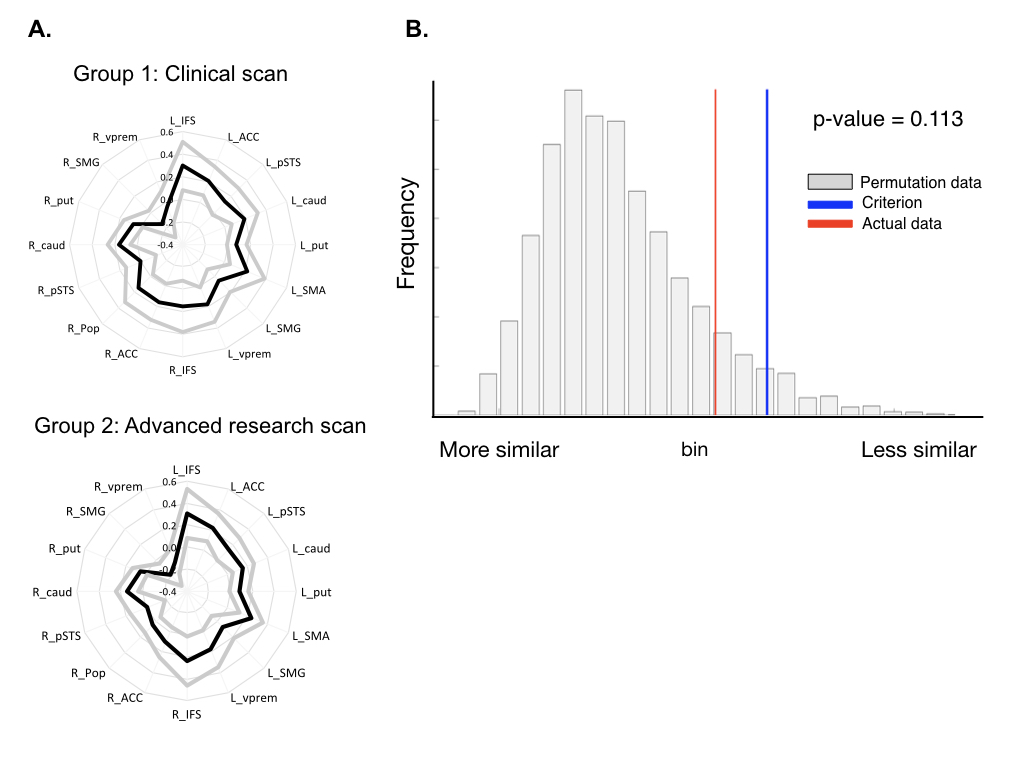
**

**Comparison of fingerprints between two groups of healthy controls. A.** Signal correlations between the left pars opercularis and target regions in the speech language network were measured during a silent word generation functional MRI task in two groups of healthy controls. Ten controls were scanned on a 3T Verio MRI machine using a clinical-grade fMRI sequence. Thirteen controls were scanned on a 3T Prisma MRI system with an ultra-fast advanced research sequence. **B**. The distance between the fingerprints of the two control groups was formally quantified using permutation testing. The Manhattan Distance test statistic did not reach criterion for statistical significance. In other words, the connectivity fingerprints for the two control groups did not differ.

**Supplementary Fig S3.**

**
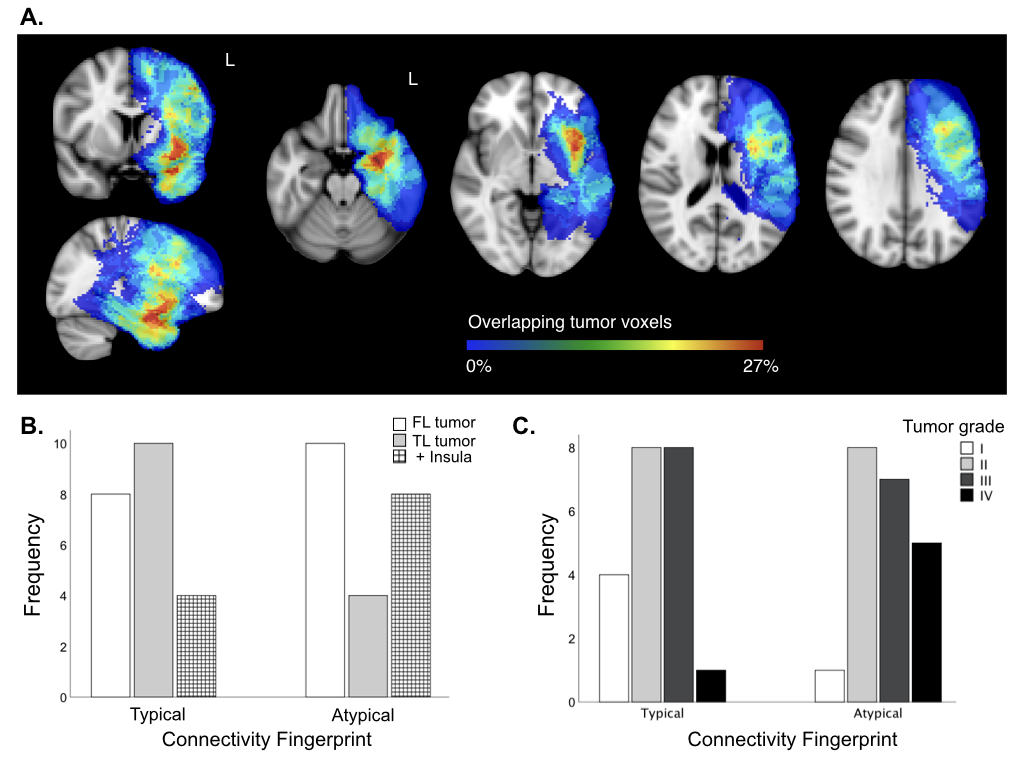
**

**Tumour overlap map and frequencies of typical vs atypical connectivity fingerprints according to tumour location and grade. A.** Overlap between tumour masks of all 44 patients, overlaid onto the MNI template brain. The percentage of overlapping voxels is depicted in the heat map, ranging from 0% (blue, voxels affected by tumour in 1 patient) to the maximum 27% (red, voxels commonly affected in 12 / 44 patients). The distribution of typical and atypical Pop connectivity fingerprints according to tumour location (**B.**) and histological grade (**C.**). While there was no difference in the incidence of typical vs atypical fingerprints according to tumour location in the temporal lobe, frontal lobe, or extending into the insula (p > 0.13), World Health Organisation histopathological grade was a significant predictor of variance in Pop connectivity (see main results and Table 3).

**Supplementary references**

Fridriksson J, Moser D, Ryalls J, Bonilha L, Rorden C, Baylis G. Modulation of frontal lobe speech areas associated with the production and perception of speech movements. J Speech Lang Hear Res 2009; 52(3): 812-9.

Gauvin HS, De Baene W, Brass M, Hartsuiker RJ. Conflict monitoring in speech processing: An fMRI study of error detection in speech production and perception. NeuroImage 2016; 126: 96-105.

Gil Robles S, Gatignol P, Capelle L, Mitchell MC, Duffau H. The role of dominant striatum in language: a study using intraoperative electrical stimulations. Journal of neurology, neurosurgery, and psychiatry 2005; 76(7): 940-6.

Jeon HA, Lee KM, Kim YB, Cho ZH. Neural substrates of semantic relationships: common and distinct left-frontal activities for generation of synonyms vs. antonyms. NeuroImage 2009; 48(2): 449-57.

Krainik A, Lehericy S, Duffau H, Capelle L, Chainay H, Cornu P*, et al.* Postoperative speech disorder after medial frontal surgery: role of the supplementary motor area. Neurology 2003; 60(4): 587-94.

Laplane D, Talairach J, Meininger V, Bancaud J, Orgogozo JM. Clinical consequences of corticectomies involving the supplementary motor area in man. J Neurol Sci 1977; 34(3): 301-14.

Lorca-Puls DL, Gajardo-Vidal A, Seghier ML, Leff AP, Sethi V, Prejawa S*, et al.* Using transcranial magnetic stimulation of the undamaged brain to identify lesion sites that predict language outcome after stroke. Brain : a journal of neurology 2017; 140(6): 1729-42.

Oberhuber M, Hope TM, Seghier ML, Parker Jones O, Prejawa S, Green DW*, et al.* Four Functionally Distinct Regions in the Left Supramarginal Gyrus Support Word Processing. Cereb Cortex 2016.

Price CJ. The anatomy of language: a review of 100 fMRI studies published in 2009. Annals of the New York Academy of Sciences 2010; 1191: 62-88.

Price CJ. A review and synthesis of the first 20 years of PET and fMRI studies of heard speech, spoken language and reading. NeuroImage 2012; 62(2): 816-47.

Tate MC, Herbet G, Moritz-Gasser S, Tate JE, Duffau H. Probabilistic map of critical functional regions of the human cerebral cortex: Broca's area revisited. Brain : a journal of neurology 2014; 137(Pt 10): 2773-82.

Wise RJ, Scott SK, Blank SC, Mummery CJ, Murphy K, Warburton EA. Separate neural subsystems within 'Wernicke's area'. Brain : a journal of neurology 2001; 124(Pt 1): 83-95.
